# Upregulation of SOX11 enhances tamoxifen resistance and promotes epithelial‐to‐mesenchymal transition via slug in MCF‐7 breast cancer cells

**DOI:** 10.1002/jcp.29629

**Published:** 2020-02-11

**Authors:** Yingsheng Xiao, Qin Xie, Qingsong Qin, Yuanke Liang, Haoyu Lin, De Zeng

**Affiliations:** ^1^ Department of Thyroid Surgery Shantou Central Hospital Shantou China; ^2^ Department of Medical Oncology Cancer Hospital of Shantou University Medical College Shantou China; ^3^ Guangdong Provincial Key Laboratory for Diagnosis and Treatment of Breast Cancer Shantou Guangdong China; ^4^ Laboratory of Human Virology and Oncology Shantou University Medical College Shantou China; ^5^ Department of Thyroid and Breast Surgery The First Affiliated Hospital of Shantou University Medical College Shantou Guangdong China

**Keywords:** breast cancer, epithelial‐to‐mesenchymal transition, SOX11, tamoxifen resistance

## Abstract

Resistance to tamoxifen remains a prominent conundrum in the therapy of hormone‐sensitive breast cancer. Also, the molecular underpinnings leading to tamoxifen resistance remain unclear. In the present study, we utilized the Gene Expression Omnibus database to identify that SOX11 might exert a pivotal function in conferring tamoxifen resistance of breast cancer. SOX11 was found to be markedly upregulated at both the messenger RNA and protein levels in established MCF‐7‐Tam‐R cells compared to the parental counterparts. Moreover, SOX11 was able to activate the transcription of slug via binding to its promoter, resulting in promoting the progress of epithelial‐to‐mesenchymal transition and suppressing the expression of ESR1. Downregulating SOX11 expression can restore the sensitivity to 4‐hydroxytamoxifen in MCF‐7‐Tam‐R cells. Survival analysis from large sample datasets indicated that SOX11 was closely related to poorer survival in patients with breast cancer. These findings suggest a novel feature of SOX11 in contributing to tamoxifen resistance. Hence, targeting SOX11 could be a potential therapeutic strategy to tackle tamoxifen resistance in breast cancer.

## Graphical abstract

SOX11 can promote the epithelial–mesenchymal transition process and enhance tamoxifen resistance in MCF‐7 cells. Hence, targeting SOX11 could be a potential therapeutic strategy to tackle tamoxifen resistance in breast cancer.

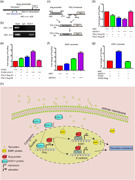

## 1 INTRODUCTION

Globally, breast cancer (BC) is the leading cancer type in women, and approximately three‐quarters of all BCs are estrogen receptor (ER) and/or progesterone receptor (PR) positive (Provenzano, Ulaner, & Chin, 2018; Waks & Winer, 2019). Endocrine therapy, particularly tamoxifen, is an essential option for the treatment of patients with ER‐positive BC (H. J. Wang, Wang, Wan, & Li, 2016). Tamoxifen significantly prolongs recurrence‐free survival (RFS) and overall survival (OS) for this subset of patients and yields clinical benefit even in those with advanced or metastatic diseases (Jager, Linn, Schellens, & Beijnen, 2015). Unfortunately, the emergence of tamoxifen therapy resistance presents a prominent challenge, as almost 30% of women experience local recurrence or distant metastasis despite the initial response (Mills, Rutkovsky, & Giordano, 2018).

The molecular underpinnings of resistance to tamoxifen are probably multifactorial but remain not fully clear. Several molecular mechanisms, including loss or modification in ER expression, activation of estrogen‐independent signaling pathways, as well as a variety of factors that regulate epithelial–mesenchymal transition (EMT) in the tumor microenvironment, are reported to be associated to tamoxifen resistance in ER‐positive tumors (Mansouri, Farahmand, Hosseinzade, Eslami, & Majidzadeh, 2017; Mills et al., 2018; Ward et al., 2013; Zhang et al., 2018). For example, Zhao et al. (2016) reported that microRNA‐221/222 downregulates the expression of the ER, and is significantly involved in tamoxifen resistance in BC. Yuan et al. (2015) demonstrated that G protein‐coupled estrogen receptor 1/estimated glomerular filtration rate/extracellular signal‐regulated kinase signaling drives the EMT and confers tamoxifen resistance in BC cells through interaction with the tumor microenvironment.

Identifying novel genes or factors that contribute to tamoxifen resistance might provide a novel avenue to overcome tamoxifen resistance for patients with hormone receptor‐positive tumors. Comparative analysis of genomic profiles from arrays of normal or cancerous tissues in predefined settings is an effective strategy to discover new targets that exert a crucial function in cancer development or drug resistance (Bertucci et al., 2016; Lanara et al., 2013). The purpose of the present study is, first, to compare the gene expression profiles of early recurrences with intermediate recurrences or early recurrences with late recurrences after tamoxifen therapy to sort out potential genes that might play a critical role in tamoxifen resistance of BC, and second, to verify the function of the candidate gene in an established tamoxifen‐resistant BC cell line.

## 2 MATERIALS AND METHODS

### 2.1 Clinical database

The expression profile GSE46222 was acquired from the Gene Expression Omnibus (GEO; http://www.ncbi.nlm.nih.gov/geo/) database. Differentially expressed genes (DEGs) between two distinct groups were screened and identified using GEO2R in the GEO database. The genes were deemed to be DEGs if |log 2‐Fold Change| ≥ 2 with *p* < .05, and the intersection among the DEGs of two groups was determined using a Venn diagram generated from an online tool (http://bioinformatics.psb.ugent.be/webtools/Venn/). The relative messenger RNA (mRNA) levels of SOX11 in a different histological grade were ascertained through the analysis of BC cases from the GOBO database (http://co.bmc.lu.se/gobo/gsa.pl). The relative mRNA levels of SOX11 in an array of Scarff Bloom and Richardson (SBR) grade, subtypes of BC were determined by bc‐GenExMiner v4.0. The relationship between the expression of SOX11 and survival was assessed by analysis in the Kaplan–Meier plotter (www.kmplot.com)

### 2.2 Cell culture and establishment of BC cell line resistant to tamoxifen

The MCF‐7 BC cell line, which was characterized by ER/PR positivity and Her‐2 negativity, was purchased from the American Type Culture Collection. The MCF‐7‐Tam‐R cells were obtained by continuous exposure to 4‐hydroxytamoxifen (Sigma‐Aldrich, St. Louis, MO) for a duration of 6 months, from 1 to 3 mM. Finally, the MCF‐7‐Tam‐R cells were maintained in medium with 3 mM 4‐hydroxytamoxifen. All the cells were maintained in Dulbecco's modified Eagle's medium (DMEM) supplemented with 10% fetal bovine serum (FBS) and were cultured in an incubator with 5% CO_2_ at 37°C.

### 2.3 Small interfering RNAs, plasmids, and transfection procedure

The small interfering RNAs (siRNAs), as shown in TableS1, were designed and synthesized by GenePharma Company (Suzhou, China). The PCMV, PCMV‐SOX11, and PCMV‐slug plasmids were purchased from Sinobiological. Lipofectamine 3000 (Life Technology, NY) was used for the transfection in the study according to the manufacturer's protocol.

### 2.4 Quantitative real‐time polymerase chain reaction

TRIzol reagent (Life Technology) was used for RNA isolation by following the manufacturer's instructions. The PrimeScript™ RT reagent kit (Takara Bio Inc., Dalian, China) was used for the reverse transcription of total RNA (1 µg) according to the manufacturer's protocols. SYBR Premix Ex Taq (Takara Bio Inc.) was used for the quantitative real‐time polymerase chain reaction (qRT‐PCR) assay on a CFX96 Real‐time PCR Detection System (Bio‐Rad, CA). The sequences of primer for qPCR are shown in Table S2. The following PCR reaction scheme was used: 5 min at 94°C followed by (30 s at 94°C, 30 s at 63°C, and 30 s at 72°C) 35×, and 10 min at 72°C.

### 2.5 Western blot analysis

Cells of 90% confluence were harvested and subsequently lysed in radioimmunoprecipitation assay buffer containing 1 mM phenylmethylsulfonyl fluoride. After 12,000*g* centrifugation for 15 min at 4°C, the protein in the supernatants was measured through the bicinchoninic acid protein assay and then the samples were stored at −80°C until use. The 8% sodium dodecyl sulfate–polyacrylamide gel electrophoresis was used to separate samples, which were then transferred onto a polyvinylidene fluoride (PVDF) membrane. After blocking in 5% fat‐free milk, the PVDF membrane was subsequently incubated with the corresponding primary antibodies (Table S3) at 4°C overnight, and followed by incubation with the appropriate secondary antibodies. Finally, the enhanced chemiluminescence reagent (Applygen, Beijing, China) was added for protein detection.

### 2.6 Cell viability assays

Cells were cultured at 5 × 10^3^ cells/well in 96‐well plates with the addition of media containing an array of concentrations (0.1 μM, 1.0 μM, 5.0 μM, 10.0 μM, 20.0 μM, 40.0 μM and 100.0 μM) of 4‐hydroxytamoxifen (4‐OH‐TAM; Sigma‐Aldrich). After 72 hr, 10 μl of Cell Counting Kit‐8 reagent (Beyotime Institute of Biotechnology, Jiangsu, China) was added in each well, and then cultured in a 5% CO_2_ and 37°C incubator for 2 hr. The corresponding absorbance of each well was read at 450 nm using a spectrophotometer (Thermo Fisher Scientific‎).

### 2.7 Immunofluorescence assay

The cells were fixed with 4% paraformaldehyde for 10 min at room temperature (RT), and subsequently treated with 0.5% Triton X‐100 for 10 min, followed by blocking for 20 min with 5% normal goat serum. Then, the cells were incubated with the primary antibody (E‐cadherin 1:100) overnight at 4°C. Next, the cells were washed with phosphate‐buffered saline three times and incubated with secondary antibodies (Alexa Fluor 488 donkey anti‐mouse immunoglobulin G [IgG] and Alexa Fluor 594 donkey anti‐rabbit IgG) at RT for 1 hr in the dark. Slides were mounted with 4′,6‐diamidino‐2‐phenylindole (Life Technology) in VECTASHIELD. Finally, stained cells were visualized, analyzed, and photographed with an immunofluorescence microscope (Carl Zeiss, Jena, Germany).

### 2.8 Luciferase reporter assay

The Dual‐Luciferase Reporter Assay System was used for the luciferase assay according to the manufacturer's instruction. Approximately, 1 × 10^5^ MCF‐7‐Tam‐R cells were plated in 24‐well plates, and then transfected with siNC, siSOX11, pGL3 Slug‐Wt promoter reporter, pGL3 slug‐Mt promoter reporter, and pRL‐SV40 using the transfection reagent Lipofectamine 3000. The luciferase activity of each group was determined 48 hr after transfection.

### 2.9 Chromatin immunoprecipitation

Cells growing at 80–90% confluence in 10‐cm dishes were fixed with 1% formaldehyde for 10 min that allowed the cross‐linking between proteins and DNA, which were prepared to perform the following chromatin immunoprecipitation (ChIP) assay utilizing the ChIP assay kit (Beyotime, Shanghai, China) according to the manufacturer's protocol.

### 2.10 Cell motility assay

Matrigel invasion chambers (BD Bioscience, CA) and 8‐mM pore size cell culture inserts (BD Bioscience) were used for the migration assay. The tumor cells (4 × 10^4^) were first seeded into the upper chamber in FBS‐free DMEM medium. DMEM supplemented with 10% FBS was added to the lower chamber. After incubation for 48 hr, the nonmigrating or noninvading cells in the top chamber were removed, and the cells on the lower surface of the membrane were stained with 0.1% crystal violet. The number of cells in five random visual fields in each chamber was counted and analyzed. The independent experiments were repeated three times.

### 2.11 Colony formation assay

Approximately, 400 transfected MCF‐7‐Tam‐R cells were plated in six‐well plates and cultured in DMEM with 10% FBS. After culturing for 2 weeks, the colonies were fixed with methanol and stained with crystal violet. Each colony exceeding 50 cells was counted.

### 2.12 Immunohistochemistry

The BC tissue microarrays from 140 female patients, none of which had received another antitumor treatment before surgery, were purchased from Shanghai Outdo Biotech (Shanghai). The SOX11 (1:200) were used as primary antibodies. The scores of intensity and extent were multiplied to generate the staining score for each section. The quantification of staining intensity represented the staining intensity (score 0, no staining; score 1, weak staining; score 2, moderate staining; sand core 3, strong staining). The percentage of positive tumor cells was recorded as follows: 0–5% as 0; 5–24% as 1, 25–49% as 2, 50–74% as 3, and >75% as 4. Subsequently, the tissue graded for each patient was based on the sum of scores: 0 (−); 1–4 (+); 5–8 (++); and >9 (+++). Cases with weighted − and + were defined as low expression, and ++ and +++ were defined as high expression.

### 2.13 Statistical analysis

All the statistical analysis in the study was performed with SPSS 23.0. Each experiment was performed in triplicate. The data were presented as the mean ± standard deviation. Student's *t* test and unpaired two‐tailed Student's *t* test were used for comparing the difference between mean values and pairs of data, respectively. A two‐sided *p* < .05 was regarded as statistically significant and indicated as *p < .05, **p < .01, and ***p < .001 (Student's *t* test) when compared with control cells.

## 3 RESULTS

### 3.1 SOX11 is identified to be differentially expressed in BC cases with a different recurrence scenario after tamoxifen therapy

**Figure 1:**
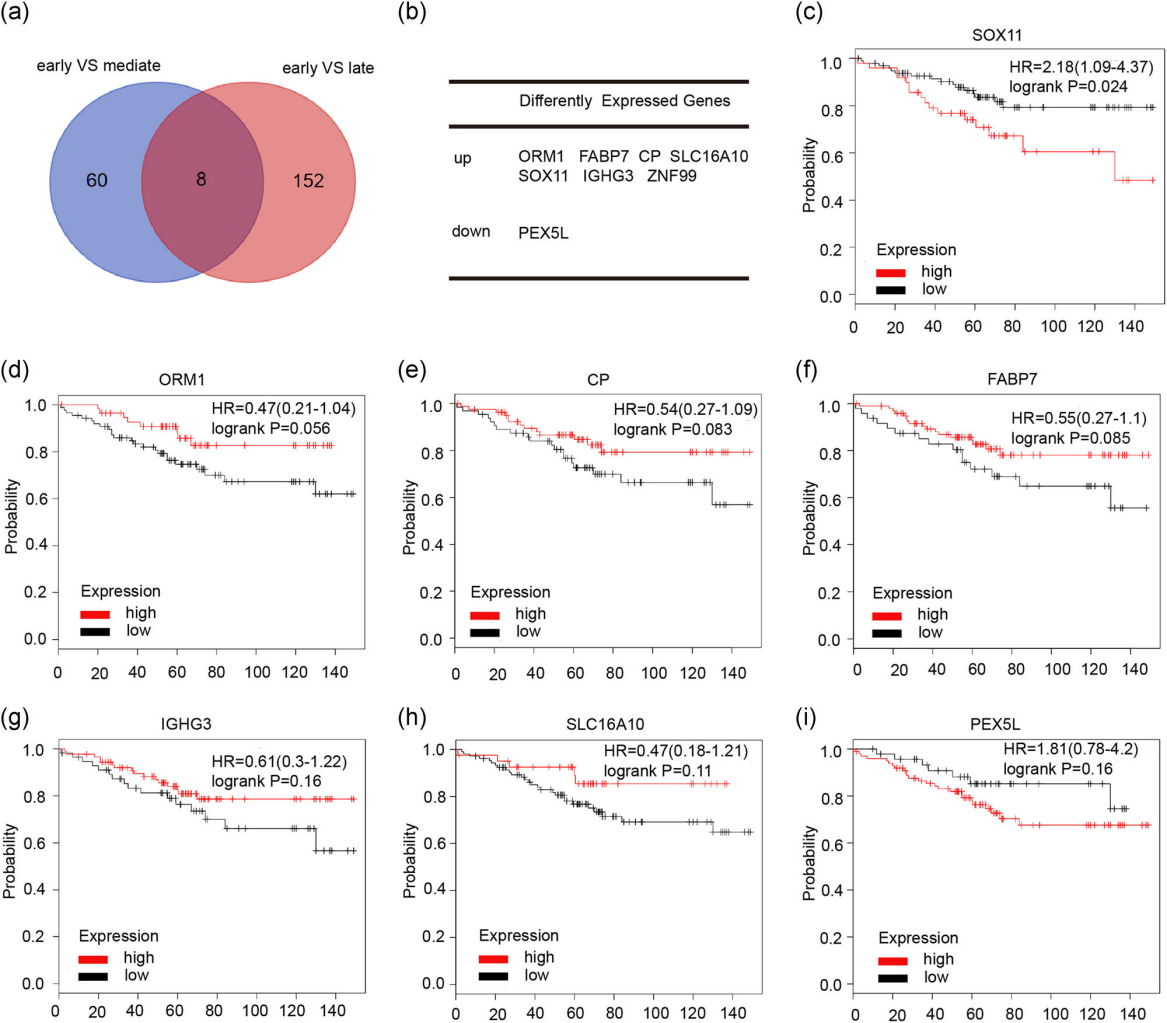
Identification of differentially expressed genes in breast cancer treated with tamoxifen. (a) Venn diagram reveals common differentially expressed genes (DEGs) in GSE46222. (b) A total of eight differentially expressed genes (seven upregulated and one downregulated genes) are displayed in the table. (c–i) The relationship between DEGs and overall survival in patients with breast cancer who received tamoxifen therapy. HR, hazard ratio

We selected three GEO datasets (GSE46222), which contained information on genomic expression for BC cases receiving tamoxifen treatment. Next, we compared gene expression profiles acquired by early recurrences compared with intermediate recurrences or early recurrences compared with late recurrences after tamoxifen therapy. Early recurrence was defined as a relapse of BC within 3 years after tamoxifen treatment, and late recurrence represents a relapse of BC 5 years after tamoxifen treatment. Intermediate recurrence refers to a relapse of BC within 3–5 years. A number of genes were identified as potential predictors at the threshold defined as |log 2‐Fold Change| ≥ 2 and *p* < .05. As shown in Figure 1a, the intersection identified a total of eight candidate genes (seven upregulated and one downregulated), which might be essential indicators of tamoxifen therapeutic efficacy in BC. Next, we utilized the public database (www.kmplot.com) to analyze the relationship between these DEGs and clinical outcomes in patients with BC who received tamoxifen treatment. As shown in Figure 1c–i, a high SOX11 mRNA level was correlated to shorter OS in all patients with BC receiving tamoxifen treatment, but other genes were not significant (the ZNF11 gene was unable to be found in this database). Therefore, these results show that SOX11 may play a vital role in patients with BC receiving tamoxifen treatment.

### 3.2 MCF‐7 cells with acquired tamoxifen resistance exhibit an EMT‐like phenotypic change and express high levels of SOX11 and slug

**Figure 2:**
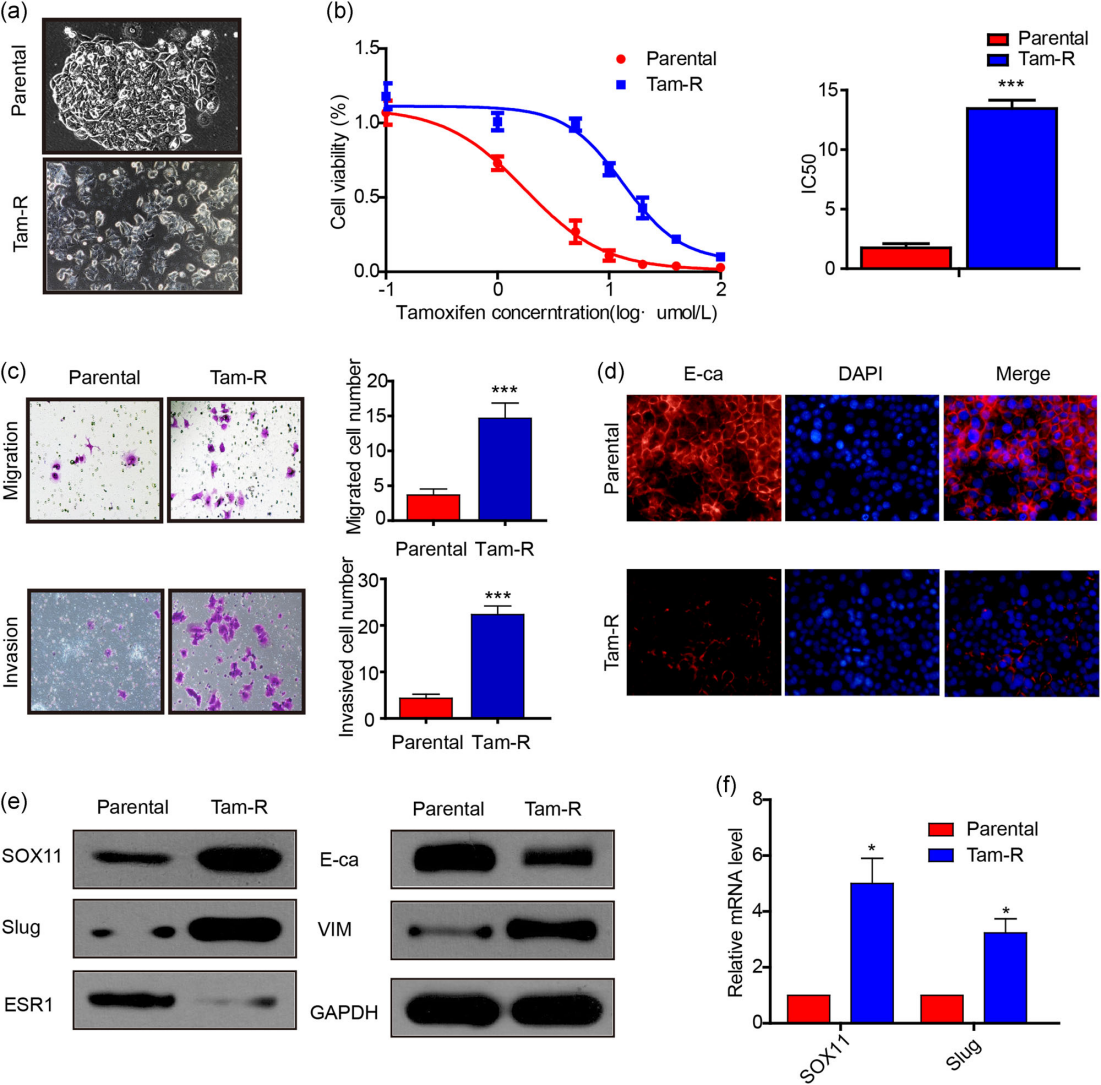
MCF‐7‐Tam‐R cells are resistant to 4‐OH‐tamoxifen treatment and show enhanced motility and invasive behaviors and express higher SOX11 and slug. (a) Morphology of MCF‐7 and MCF‐7‐Tam‐R cells. (b) Cell viability analysis of MCF‐7 and MCF‐7‐Tam‐R cells after treatment with 4‐OH‐tamoxifen. (c) The cell invasion and migration capacity were examined using a Transwell assay in MCF‐7 and MCF‐7‐Tam‐R cells. (d) Immunofluorescence of MCF‐7‐Tam‐R cells and their parental cells stained with anti‐E‐ca (red signal) antibody, DAPI (blue signal). (e) Relative protein levels of SOX11, slug, ESR1, E‐ca, and vimentin were detected by western blot analysis in MCF‐7 and MCF‐7‐Tam‐R cells. (f) The mRNA levels of SOX11 and slug in MCF‐7 and MCF‐7‐Tam‐R cells were determined by real‐time RT‐PCR. DAPI, 4′,6‐diamidino‐2‐phenylindole; mRNA, messenger RNA; RT‐PCR, real‐time‐polymerase chain reaction

The above results suggested that SOX11 may play a vital role in tamoxifen resistance in BC, and we, therefore, established a tamoxifen‐resistant MCF‐7‐Tam‐R cell line by exposing the cells to tamoxifen for subsequent mechanistic studies. We could observe that the MCF‐7 cells were growing in tightly packed cobblestone‐like clusters and demonstrated features typical of epithelial cells. In contrast, MCF‐7‐Tam‐R cells appeared to lose tight cell–cell contact and displayed a fibroblast‐like morphology (Figure 2a). Next, the Cell Counting Kit‐8 assay was performed to determine the IC50 value to validate tamoxifen resistance in MCF‐7‐Tam‐R cells. The results showed that the parental MCF‐7 cells were 1.760 ± 0.35 μM and the MCF‐7‐Tam‐R cells were 13.46 ± 0.7024 μM (Figure 2b). To test whether the MCF‐7‐Tam‐R cells displayed increased migratory and invasive properties than those of the parental MCF‐7 cells, a transwell assay was performed. We found that MCF‐7‐Tam‐R cells demonstrated significantly increased migratory and invasive abilities compared with their parental counterparts (*p* < .001; Figure 2c).

Next, we examined whether MCF‐7‐Tam‐R cells exhibited molecular changes in EMT. We found that decreased expression of E‐cadherin in MCF‐7‐Tam‐R cells, as compared with parental cells, was detected by immunofluorescence (Figure 2d). The expression of E‐cadherin and ESR1 decreased, while the expression of vimentin and slug increased in MCF‐7‐Tam‐R cells in western blot analysis (Figure 2e,f). More surprisingly, the SOX11 protein was also upregulated in MCF‐7‐Tam‐R cells. The gray value measurement and statistical analysis of western blot are shown in Figure S2. These results suggested that SOX11 upregulation and EMT process in MCF‐7‐Tam‐R cells were closely related to tamoxifen resistance in BC.

### 3.3 Inhibition of SOX11 downregulates slug expression and reversed EMT in MCF‐7‐Tam‐R cells

**Figure 3:**
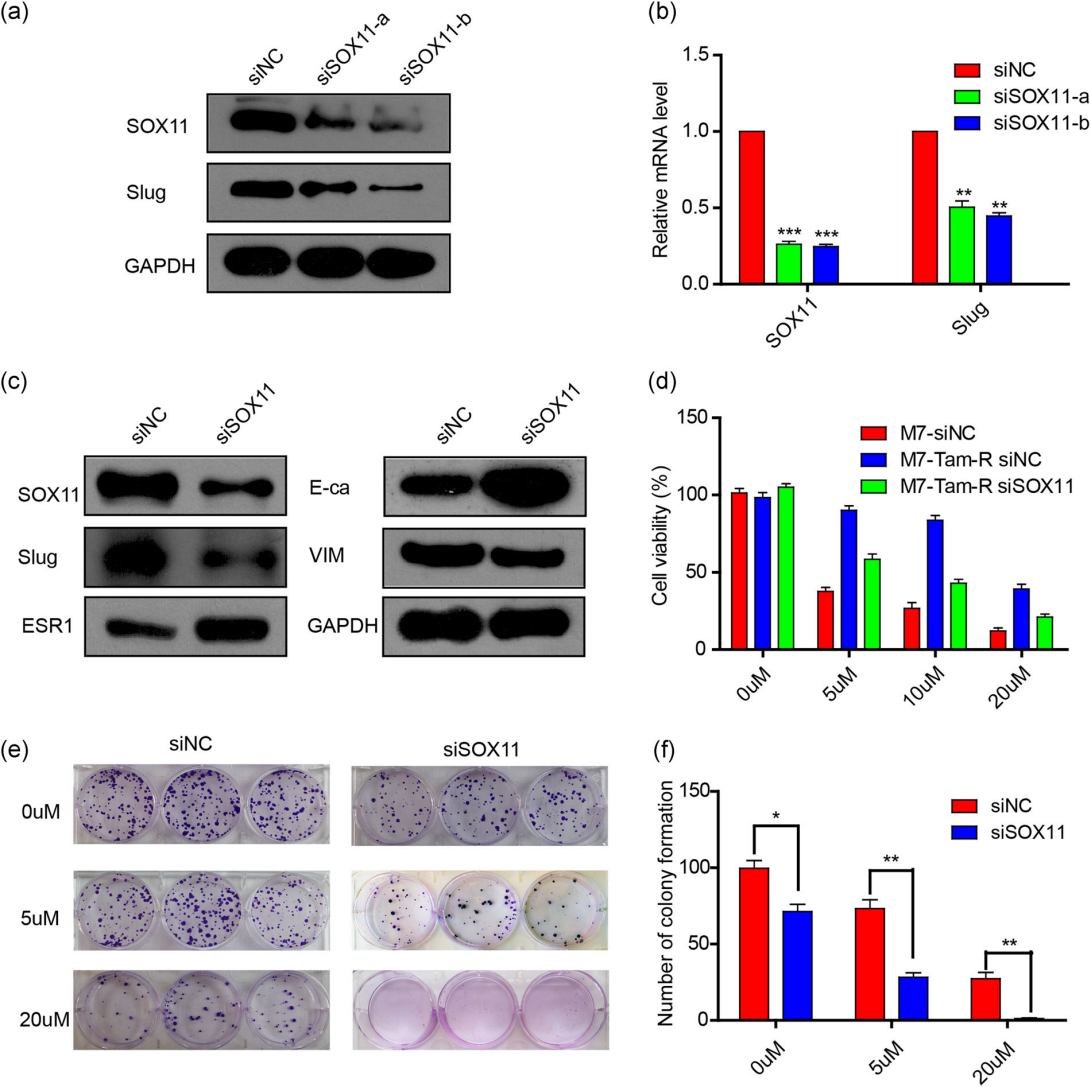
SOX11 knockdown in MCF‐7‐Tam‐R cells decreases slug level and reversed the EMT process. (a and b). MCF‐7‐Tam‐R cells were transfected with two different sequences of SOX11 siRNAs. (c) Western blot analysis confirmed that SOX11 knockdown in MCF‐7‐Tam‐R cells could decrease the expression of slug and reverse the EMT process. (d) Cell viability was examined using the CCK‐8 assay after treatment with four different concentrations of 4‐OH‐tamoxifen for 72 hr in siNC transfected MCF‐7 cells, siNC transfected MCF‐7‐Tam‐R cells, and siSOX11 transfected MCF‐7‐Tam‐R cells. (e and f). Colony numbers were counted in the MCF‐7‐Tam‐R cells and siSOX11 transfected MCF‐7‐Tam‐R cells. CCK‐8, Cell Counting Kit‐8; EMT, epithelial–mesenchymal transition; mRNA, messenger RNA; siNC, siRNA negative control; siRNA, small interfering RNA

To clarify the exact function of SOX11 in BC, we downregulated its expression in MCF‐7‐Tam‐R cells via transfection of SOX11 siRNAs. Our data showed that the SOX11 siRNAs significantly decreased both the mRNA and protein expression levels of SOX11. Because siSOX11‐b demonstrated more efficiency than siSOX11‐a in the inhibition of SOX11, we used siSOX11‐b for the subsequent experiments (Figure 3a,b). As shown in Figure 3c, downregulation of SOX11 could reduce the expression of slug, leading to a marked decline in the expression of vimentin, while an elevation in the expression of ESR1 and E‐cadherin. Next, we investigated whether inhibition of SOX11 could reverse the sensitivity to tamoxifen in MCF‐7‐Tam‐R cells. We transfected siSOX11 or siNC in MCF‐7‐Tam‐R cells and exposed them to different concentrations of 4‐OH‐TAM. It was found that, as compared with the control group, cell viability in the siSOX11 group declined by 40% at 5 μmol/L 4‐OH‐TAM, 50% at 10 μmol/L 4‐OH‐TAM, and 50% at 20 μmol/L 4‐OH‐TAM, respectively (Figure 3d). Furthermore, depletion of SOX11 could inhibit colony formation ability, particularly in the cells treated with tamoxifen (Figure 3e,f). Thus, these findings indicated that SOX11 was an essential contributor to tamoxifen resistance, partly through regulating slug expression and reversing EMT in MCF‐7‐Tam‐R cells.

### 3.4 SOX11 induces EMT and tamoxifen resistance in MCF‐7 cells

The above results indicated that SOX11 inhibition can suppress the EMT process in MCF‐7‐Tam‐R cells. To further investigate the effects of SOX11 on EMT, a series of experiments were performed as follows. The western blot analysis assay showed that the expression of E‐cadherin and ESR1 decreased, while the expression of vimentin and slug increased after being transfected by PCMV‐SOX11 plasmids in MCF‐7 cells (Figure S1a). The SOX11 overexpression also increased MCF‐7 cell migration and invasion capacity (Figure S1b,c). Moreover, elevated SOX11 was sufficient to promote tamoxifen resistance (Figure S1d). Together, these findings demonstrate that SOX11 can induce EMT and promote tamoxifen resistance in MCF‐7 cells.

### 3.5 SOX11 promotes the EMT process mediated by slug

**Figure 4:**
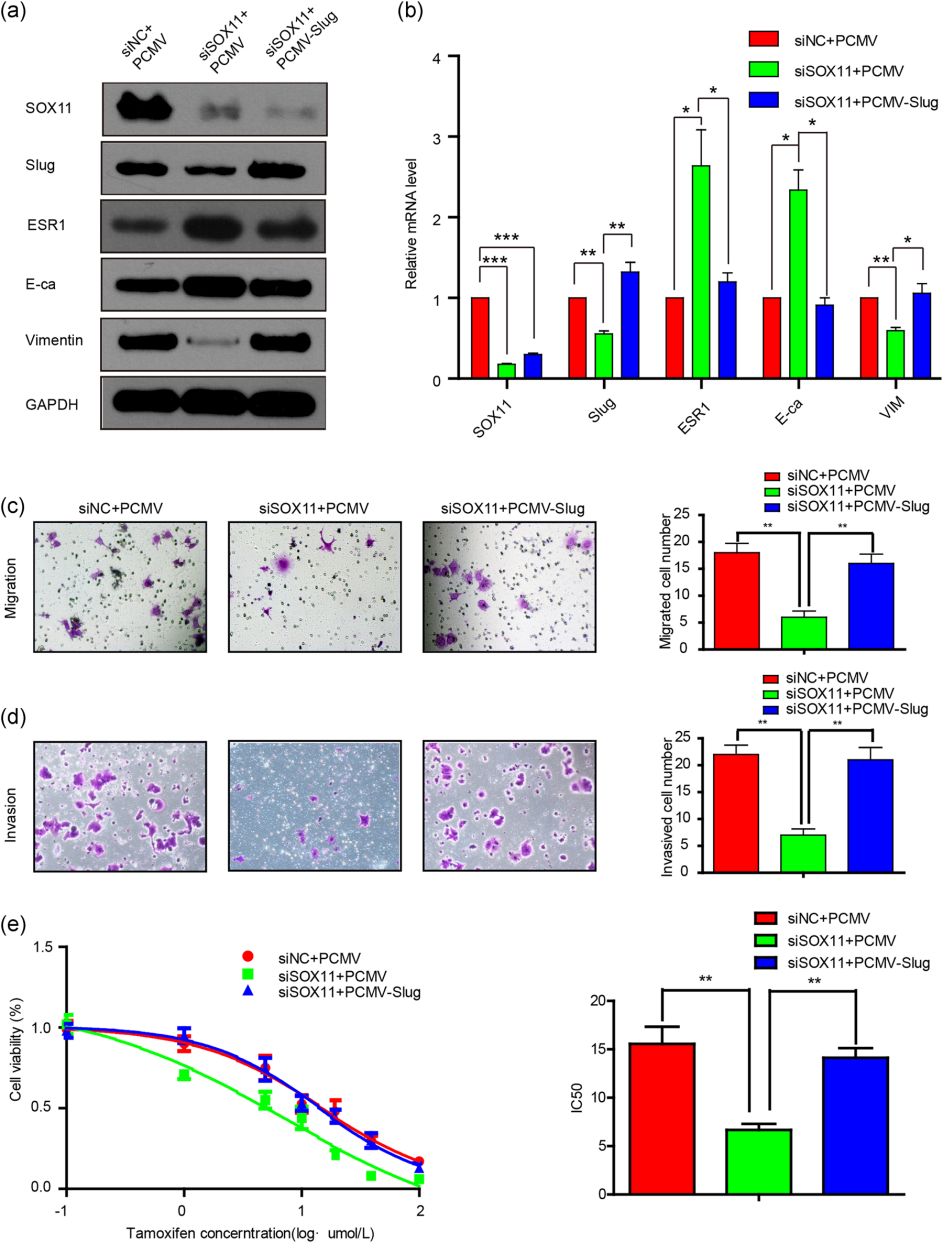
SOX11 regulates the EMT process via slug. (a and b) MCF‐7‐Tam‐R cells were simultaneously transfected with siSOX11 and PCMV‐slug and the levels of SOX11, slug, and the EMT‐related proteins were analyzed by western blot analysis and real‐time RT‐PCR. (c and d) The cell invasion and migration capacity were examined using a Transwell assay in MCF‐7‐Tam‐R cells, cotransfected with siSOX11 and PCMV‐slug. (e) Cell viability analysis of MCF‐7‐Tam‐R cells, cotransfected with siSOX11 and PCMV‐slug after treatment with 4‐OH‐tamoxifen. EMT, epithelial–mesenchymal transition; mRNA, messenger RNA; RT‐PCR, real‐time‐polymerase chain reaction; siNC, siRNA negative control

It has been reported that slug can repress the transcription of CDH1 (encoding E‐cadherin) by binding to its E‐box elements (Hajra, Chen, & Fearon, 2002). To further confirm that SOX11 could promote the EMT process via slug, we performed the following rescue experiments of cotransfecting siSOX11 and PCMV‐slug into BC cells. RT‐PCR and western blotting assays revealed that the upregulated expression of ESR1 and E‐cadherin induced by siSOX11 was significantly attenuated by overexpression of slug in MCF‐7‐Tam‐R cells, as compared with cells cotransfected with PCMV. Conversely, vimentin expression was reproducibly increased after overexpression of slug (Figure 4a,b). Next, we performed transwell assays to determine the influence of SOX11 and slug on cell motility and invasion in MCF‐7‐Tam‐R cells. As expected, slug was able to partially reverse the decline of motility and invasion ability caused by SOX11 depletion (Figure 4c,d). Furthermore, slug could partially restore enhanced tamoxifen sensitivity caused by downregulation of SOX11 (Figure 4e). These results suggested that SOX11 could promote the EMT process and tamoxifen resistance by modulating the expression of slug.

### 3.6 SOX11 enhances slug transcription through binding to its promoter

**Figure 5:**
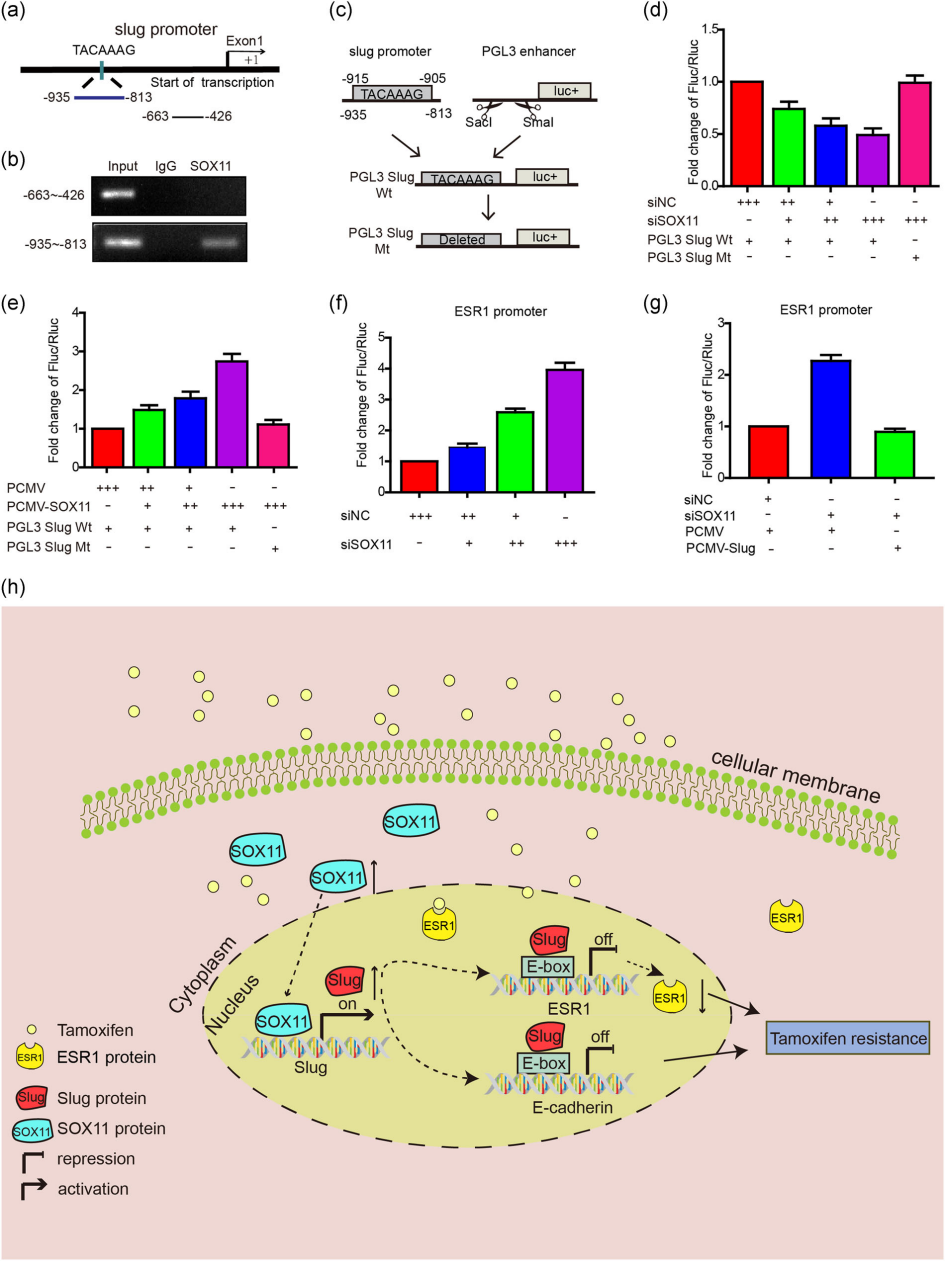
SOX11 regulates the expression of slug by binding to the slug promoter. (a) The human slug promoter sequence. (b) SOX11 binding on the slug promoter is shown using the ChIP assay. (c) The schematic diagram of constructs with a luciferase reporter plasmid. (d) Luciferase assays were performed in MCF‐7‐Tam‐R cells by cotransfection with siNC, siSOX11, PGL3 slug Wt, or PGL3 slug Mt and luciferase activity was normalized to the renilla minimal reporter. (e) Luciferase assays were performed in MCF‐7 cells by cotransfection with PCMV, PCMV‐SOX11, PGL3 slug Wt, or PGL3 slug Mt and luciferase activity was normalized to the renilla minimal reporter. (f and g) Luciferase assays were performed in MCF‐7‐Tam‐R cells by cotransfection with siSOX11, ESR1 promoter with or without PCMV‐slug, and luciferase activity was normalized to the renilla minimal reporter. (h) Schematic diagram showing enhanced SOX11‐mediated tamoxifen resistance by modulating slug expression. ChIP, chromatin immunoprecipitation; IgG, Immunoglobulin G; siNC, siRNA negative control

The above results provided critical evidence that SOX11 was capable of regulating slug both at the mRNA and protein levels. We, therefore, speculated that SOX11 might act as a transcription factor to transcriptionally regulate slug. It has been reported that SOX11 transcriptionally regulated downstream genes via binding to the promoter site of target genes (A/T) (A/T) CAA (A/T) G. After analyzing a roughly 2,000 bp fragment upstream of the translation start site in the slug gene, we identified one potential SOX11‐binding element (TACAAAG). To determine whether SOX11 could bind to the promoter of slug, we designed a series of sequence primers (−935 to −813), which included the SOX11‐binding sites (TACAAAG) and negative control (−663 to −426; Figure 5a). ChIP assay was subsequently performed to determine whether SOX11 was able to bind to putative SOX11‐binding sites within the promoter region of slug. We found that the SOX11 antibody could bind to the slug promoter region containing putative SOX11 binding sites, while the control IgG and the negative control did not show a similar result (Figure 5b). These findings clearly demonstrated that SOX11 directly bound to the slug promoter.

To determine whether SOX11 could drive slug promoter activity, we cloned a luciferase reporter vector that contained the SOX11‐binding sites (pGL3‐SOX11 Wt), along with a mutant vector in which the SOX11‐binding site was deleted (pGL3‐SOX11 Mt; Figure 5c). When cotransfected with 10, 20, and 40 pmol siSOX11, slug promoter activity decreased dose dependently in the MCF‐7‐Tam‐R cell line; however, SOX11 knocking down did not attenuate the mutant slug promoter activity (Figure 5d). In addition, the luciferase reporter assay was performed in MCF‐7 cells. The overexpressed SOX11 could increase slug promoter activity, while the mutant slug reduced this effect (Figure 5e).

The above results revealed that SOX11 could transcriptionally regulate slug expression by binding to the slug promoter. Moreover, some studies have proved that slug can suppress ESR1 expression by binding to its E‐box element. We speculated that SOX11 could transcriptionally suppress ESR1 by upregulating slug expression. First, we cloned the ESR1 promoter (928 bp upstream of exon1, extending to +72 bp) before a luciferase reporter gene. When cotransfected with 10, 20, or 40 pmol siSOX11, the ESR1 promoter activity increased in a dose‐dependent manner in the MCF‐7‐Tam‐R cell line (Figure 5f). Overexpression of slug could abrogate this increase (Figure 5g). These results indicated that SOX11 repressed ESR1 by upregulating slug.

### 3.7 Higher mRNA level of SOX11 is associated with the high‐pathological grade and poor prognosis

**Figure 6:**
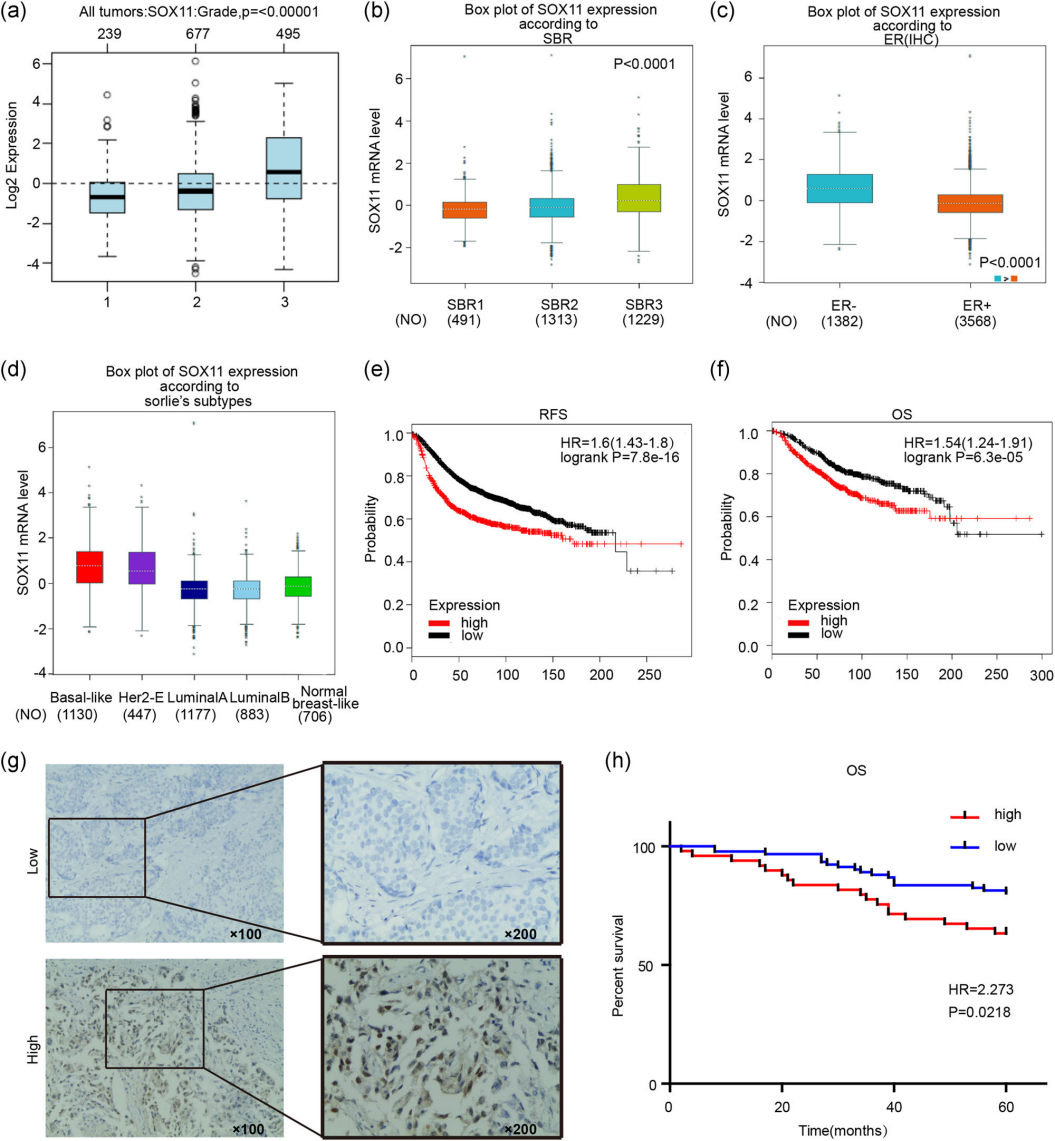
The prognostic values of SOX11 in patients with breast cancer (BC). (a) SOX11 expression in BC tissues of different histological grades. (b) SOX11 expression in BC tissues of different Scarff Bloom and Richardson grade status (SBR). (c) The expression of SOX11 in ER‐positive and ER‐negative tumors. (d) The expression of SOX11 in several subtypes of patients with BC. (e and f) The high mRNA level of SOX11 was significantly associated with poorer OS and RFS in all patients with BC. (g) Immunohistochemical analysis of SOX11 in patients’ tissues. (h) The high protein level of SOX11 was significantly associated with poorer OS in all patients with BC. ER, estrogen receptor; HR, hazard ratio; IHC, immunohistochemistry; mRNA, messenger RNA; OS, overall survival; RFS, recurrence‐free survival

The above results indicated that SOX11 was closely related to tamoxifen resistance in BC cell lines. We further examined the relationship between the level of SOX11 and tumor grade, as well as survival in BC. Through extensive analysis in a public database with BC cases, we found that the level of SOX11 was markedly higher in high‐ than low‐pathological grade BC (*p* ≤ .00001; Figure 6a). In addition, according to the SBR grade status criterion, a higher mRNA level of SOX11 was correlated to a more advanced SBR grade (*p* < .0001; Figure 6b). Correlation analysis in bc‐GenExMiner v4.0 demonstrated that mRNA levels of SOX11 were higher ER‐negative than ER‐positive tumors (Figure 6c). Moreover, mRNA levels of SOX11 were significantly higher in basal‐like and HER‐2 subtypes than those in Luminal A and Luminal B subtypes of BC (Figure 6d). Next, we investigated the prognostic effect of SOX11 in patients with BC through survival analysis in the Kaplan–Meier plotter. It was found that elevated mRNA levels of SOX11 were significantly associated with shorter RFS (hazard ratio [HR] = 1.6, *p* = 7.8e−16; Figure 6e) and shorter OS (HR = 1.54, p = 6.3e−05; Figure 6f) in patients with BC. Thus, SOX11 appears to be a pivotal biomarker for predicting tumor grade and survival in BC.

Moreover, we utilized tissue microarrays to analyze the relationship between SOX11 protein expression and clinical‐pathological features. There were 49 cases with high SOX11 expression and 91 cases with low SOX11 expression (Figure 6g). The SOX11 expression in the ER‐negative group (*p* = .001) and PR‐negative group (*p* = .003) were significantly higher than those in the positive group. None of the other clinical‐pathological parameters analyzed, including lymph node metastasis, tumor size, Her‐2 or Ki‐67, were significantly related to SOX11 expression (*p* > .05; Table S4). Survival analysis showed that high SOX11 protein expression was associated with poor 5‐year OS in patients with BC (HR = 2.273, *p* = .0218; Figure 6h).

## 4 DISCUSSION

Intrinsic or subsequently acquired resistance to endocrine therapy continues to be a major obstacle in the therapy for hormone receptor‐positive BC (Szostakowska, Trebinska‐Stryjewska, Grzybowska, & Fabisiewicz, 2019). Although current treatment that targets the phosphatidylinositol‐3‐kinase/protein kinase B/mammalian target of rapamycin and cyclin D/cyclin‐dependent kinases 4 and 6 signaling pathways, usually in combination with aromatase inhibitors or fulvestrant, have markedly improved the survival of this subset of patients (Araki & Miyoshi, 2018; Layman, 2019), it remains imperative to identify novel potent modulators that regulate the cellular response to endocrine therapy in BC. Herein, through conducting an in‐depth analysis of datasets derived from the GEO database, we found that SOX11 was differentially expressed in BC cases in different recurrence scenarios treated with tamoxifen. Furthermore, the high mRNA level of SOX11 was associated with poor survival. These findings provide important clues and indicate a significant value to further explore the exact function of SOX11 conferring tamoxifen resistance in hormone‐sensitive BC.

SOX11 belongs to the SoxC group in the Sox transcription factors family (Hoser et al., 2008). It has been reported to be abnormally expressed in multiple types of human cancers, including prostate, gastric, and BC, and so forth; however, its exact influence on the progression of these cancers remains unclear. In prostate cancer, elevated SOX11 could suppress the invasive and migrative abilities of prostate cancer cells in vitro (Yao et al., 2015), and SOX11 promoter hypermethylation was correlated to adverse clinicopathological properties (Pugongchai, Bychkov, & Sampatanukul, 2017). In BC, Shepherd et al. (2016) reported that the SOX11 transcription factor was a crucial modulator of cell proliferation and mobility in basal‐like BC and high SOX11 was linked to a poor prognosis. Zvelebil et al. (2013) proved that SOX11 could promote the apoptosis of BC cells via regulating the level of cleaved caspase‐3. L. Wang et al. (2018) reported that miR‐211‐5p can inhibit the SOX11 expression, leading to the inhibition of proliferation, invasion, and migration in papillary thyroid cancer cells. More recently, Piva et al. (2014) reported that Sox2, another member of the Sox family, was able to promote tamoxifen resistance in BC cells. However, until now, the role of SOX11 in contributing to drug resistance in malignancies, particularly resistance to tamoxifen in BC, has not been reported.

In the established tamoxifen‐resistant BC cell line MCF‐7‐Tam‐R, which manifested an EMT‐like phenotypic change, we found that the expressions of both SOX11 and slug were significantly higher than those in the parental counterparts. Previous studies have demonstrated that tamoxifen resistance BC cells usually exhibited a spindle shape and more elongated morphology, whereas control cells tend to present with a cuboidal shape (Won, Lee, Oh, Nam, & Lee, 2016). This phenomenon was also observed in this study, indicating a tight link between tamoxifen resistance and features of EMT, as well as the involvement of SOX11 and slug as potential regulators or mediators in these processes.

Slug is recognized to be one of the key transcription factors that modulate the EMT program and was recently identified to be an important tamoxifen resistance inducer in BC (Shao et al., 2015). Kim, Lee, Oh, Nam, and Lee (2015) reported that slug significantly increased in tamoxifen‐resistant cells. More interestingly, it has been found that acquisition of the EMT phenotype was closely associated with tamoxifen resistance in BC cells (Liang et al., 2017; Yuan et al., 2015). This study demonstrated that SOX11 was able to promote the EMT process mediated by slug. Consistently, inhibition of SOX11 would downregulate slug expression and reverse EMT in MCF‐7‐Tam‐R cells. A similar change was also observed in the migratory and invasive capacity of MCF‐7‐Tam‐R cells accompanying the alteration of SOX11 expression.

A number of studies have proposed that inhibition of slug could reverse resistance to tamoxifen therapy in BC cells (Adhikary et al., 2014). Geng et al. (2016) reported that curcumin represses 4‐OH‐TAM resistance in BC cells via modulating the slug/hexokinase 2 signaling pathway. In the present study, downregulating SOX11 expression can restore the sensitivity to 4‐OH‐TAM in MCF‐7‐Tam‐R cells. Thus, these findings indicated that SOX11 was an essential mediator of EMT and tamoxifen resistance by modulating slug expression. Mechanistically, the ChIP and luciferase reporter assays consistently suggested that SOX11 enhanced slug transcription by binding to its promoter, resulting in downregulation of the ER expression. These results were supported by Li et al. (2015) study, which reported that slug promotes cancer progression by directly regulating the ERα pathway. Bai et al. (2017) also proved that slug transcriptionally inhibits ERα expression by recruiting LSD1 to the ESR1 promoter in BCs.

In addition, utilizing the publicly accessible online database with 6,234 BC cases, we found that the mRNA level of SOX11 was notably higher in high‐ rather than low‐pathological grade BC. In bc‐GenExMiner v4.0, it was found that SOX11 mRNA levels were markedly higher in ER‐negative than ER‐positive tumors. Moreover, higher mRNA levels of SOX11 were detected in basal‐like and HER‐2 subtypes than those in luminal A and luminal B subtypes of BC, which implies that SOX11 could be an important marker that characterizes the ER‐negative tumor. Immunohistochemistry analysis of BC tissue microarrays corroborated the fact that high SOX11 protein expression was also associated with ER‐negative tumors and poor survival. These findings consistently suggested that SOX11 might act as an oncogenic driver in the pathogenesis and development of BC.

In summary, this study identifies that the SOX11 gene exerts a considerable function in conferring tamoxifen resistance in BC. Inhibition of SOX11 can restore the sensitivity to tamoxifen, and reverse the EMT process in MCF‐7 cells. A high level of SOX11 is positively associated with high‐pathological grade and ER‐negative tumors and predicts poor survival in patients with BC. It is, therefore, expected that the suppression of SOX11 will be a potent therapeutic strategy to tackle tamoxifen resistance in hormone receptor‐positive BC.

## CONFLICT OF INTERESTS

The authors declare that there are no conflict of interests.

## AUTHOR CONTRIBUTIONS

Y. X. and D. Z. conceived and designed the project. Q. Q., Y. L., and H. L. analyzed the data and prepared the figures. Y. X. and Q. X. performed the experiments. Y. X. and D. Z. wrote the manuscript. D. Z. approved the final version to be submitted. All authors read and approved the manuscript and agree to be accountable for all aspects of the research in ensuring that the accuracy or integrity of any part of the work is appropriately investigated and resolved.

## Supporting information

Supplementary information

## Data Availability

Research data are not shared.
