# Supplementary material for: Upregulation of SOX11 enhances tamoxifen resistance and promotes epithelial‐to‐mesenchymal transition via slug in MCF‐7 breast cancer cells
Source: J Cell Physiol. 2020 Feb 11;235(10):7295–308. doi: 10.1002/jcp.29629 (PMC7496966; doi:10.1002/jcp.29629)
Supplement: Supplementary file 1 — Supplementary information [file JCP-235-7295-s001.doc]

**Supplementary**

**Table S1. Oligonucleotide sequences for siRNA constructs**

| Small interference RNAs | sense（5'-3'） |
| --- | --- |
| siSOX11-a | 5′-CACUCAUAACGUUCCAUGU (dTdT)- 3′′ |
| siSOX11-b | 5′-CAGGUAUGGGACACCUAGU(dTdT)- 3′ |

**Table S2. Primers used in real-time PCR**

| Gene | Forward primer | Reverse primer |
| --- | --- | --- |
| SOX11 | 5′-ATCAAGCGGCCCATGAAC -3′ | 5′-TGCCCAGCCTCTTGGAGAT-3′ |
| Slug | 5′-AGATGCATATTCGGACCCACA-3′ | 5′-CCTCATGTTTGTGCAGGAGAG-3′ |
| GAPDH | 5′-TGGACTCCACGACGTACTCAG-3′ | 5′-ACATGTTCCAATATGATTCCA-3′ |

**Table S3. Antibodies used in this study**

| Antibody | Cat. # | Company | Con. Species |
| --- | --- | --- | --- |
| Anti-Sox11 | ab170916 | Abcam (Danvers,MA,USA) | 1:2000 Rabbit |
| Anti-Slug | ab51772 | Abcam (Danvers,MA,USA) | 1:1000　 Mouse |
| Anti-E-ca | 3195 | Cell Signaling Technology (Danvers,MA,USA) | 1:1000　 Rabbit |
| Anti-VIM | 5741 | Cell Signaling Technology (Danvers,MA,USA) | 1:1000　 Rabbit |
| Anti-ESR1 | 13258 | Cell Signaling Technology (Danvers,MA,USA) | 1:1000　 Rabbit |
| Anti-GAPDH | TA-08 | ZhongshanJinqiao（Beijing,China) | 1:3000 Mouse |

**Table S4. Clinicopathological characteristics of the breast cancer patients according to SOX11 expression**

| **Clinicopathologic parameters** | **SOX11** | | ***P* value** |
| --- | --- | --- | --- |
|  | **Negative** | **Positive** |  |
| **Lymph node** |  |  | 0.552 |
| Negative | 47 | 23 |  |
| Positive | 43 | 26 |  |
| **Tumor size** |  |  |  |
| T0-T2 | 80 | 41 | 0.775 |
| T3-T4 | 10 | 6 |  |
| **ER** |  |  |  |
| Negative | 19 | 32 | 0.001 |
| Positive | 65 | 16 |  |
| **PR** |  |  | 0.003 |
| Negative | 44 | 36 |  |
| Positive | 43 | 11 |  |
| **HER2** |  |  | 0.069 |
| Negative | 66 | 29 |  |
| Positive | 23 | 20 |  |
| **Ki67** |  |  | 0.087 |
| Low | 60 | 26 |  |
| High | 27 | 22 |  |


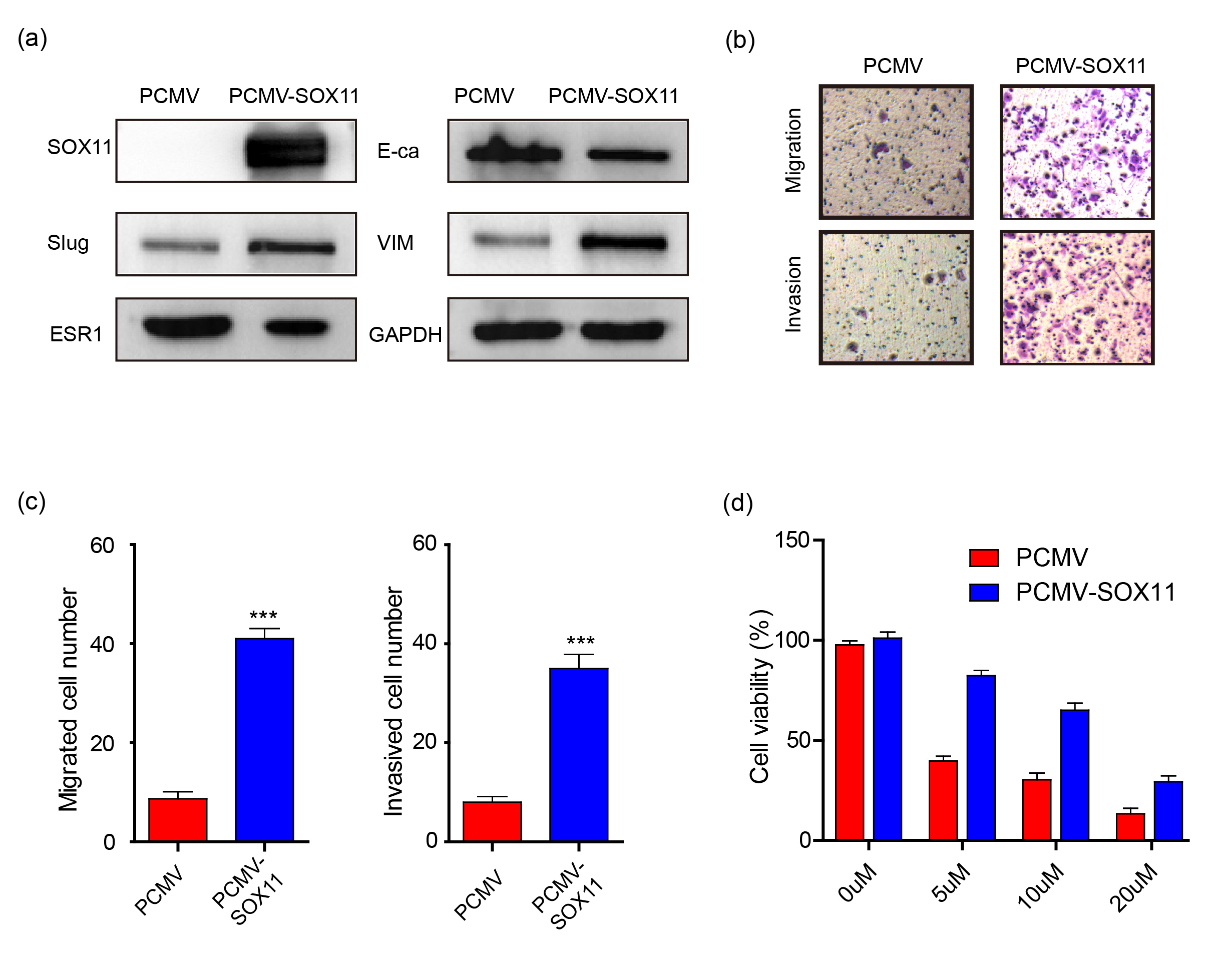


**Figure S1**. SOX11 overexpression in MCF-7 cells activates the EMT process and tamoxifen resistance.(a). The SOX11, Slug and EMT-related protein were assessed by Western blot analysis in MCF-7 cells transfected with PCMV-SOX11 or PCMV. (b)- (c). The cell invasion and migration capacity were examined using the Transwell assay in MCF-7 cells transfected with PCMV-SOX11 or PCMV. (d). Cell viability analysis of MCF-7 cells transfected with PCMV-SOX11 or PCMV, 72 h after treatment with 4-OH-tamoxifen.

**
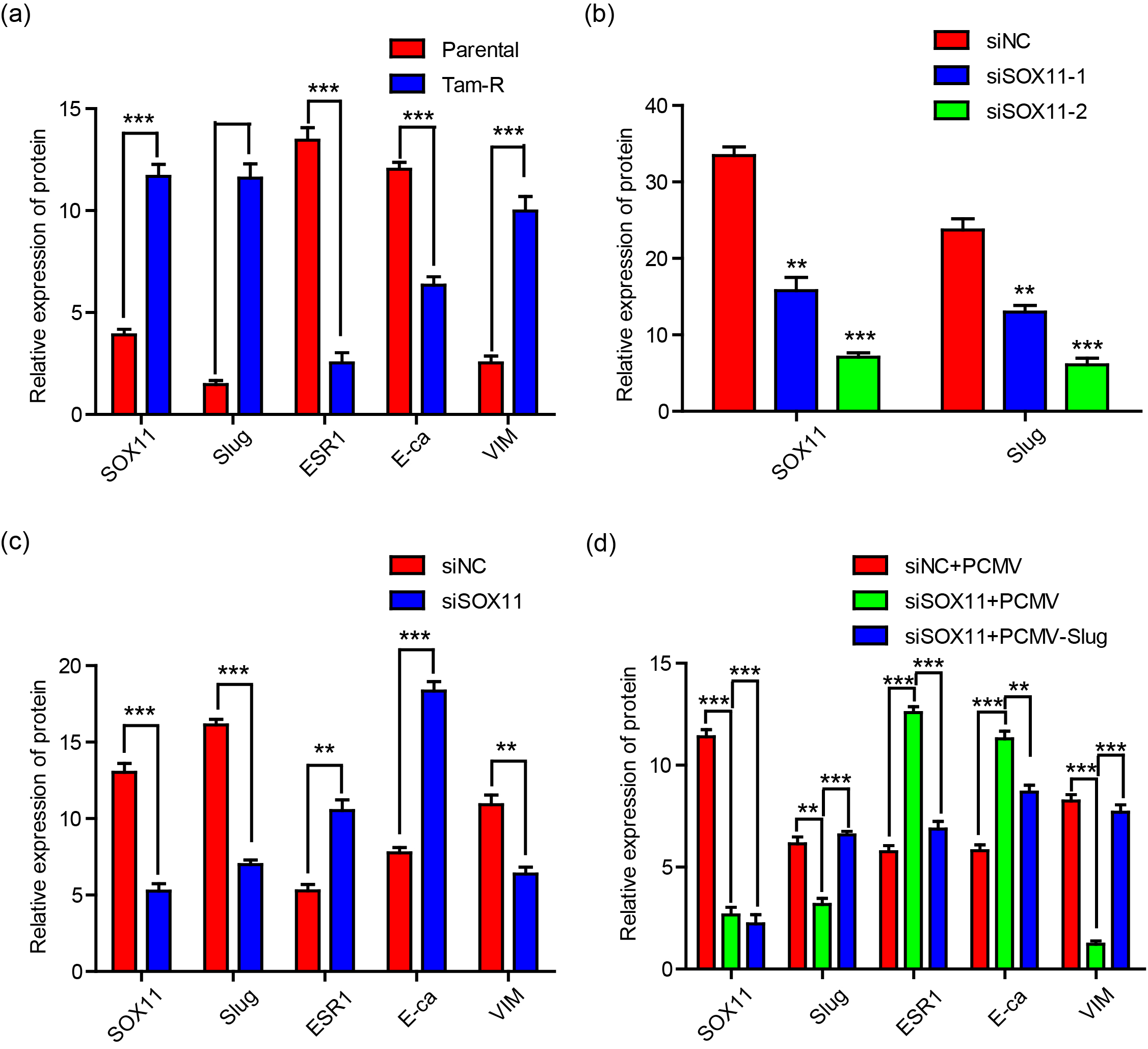
**

**Figure S2.** Gray value measurement and statistical analysis of Western-blotting in Figure 2.（e）Figure 3（a and c) and Figure 4（a）.
